# Assessing Cognitive Risk Factors in a Young Population: Findings from a Cognitive Neurology Service of Argentina

**DOI:** 10.1002/alz.086817

**Published:** 2025-01-09

**Authors:** Waleska Berrios, Agustin Perez, Maria Jose Garcia Basalo, Milagros Segui, Solange Deppleler, Maria Mercedes Garcia Basalo, Flavia Turner, Mariana Majul, Betsabe León, Agustina Melnitzky, Laura Chinen, Marcos Ojea Quintana, Angel Golimstok, Ximena Giordano, Maria Cecilia Fernandez

**Affiliations:** ^1^ Hospital Italiano de Buenos Aires, Buenos aires Argentina; ^2^ Hospital Italiano de Buenos Aires, Buenos Aires Argentina; ^3^ Hospital Italiano de Buenso Aires, Buenos Aires Argentina

## Abstract

**Background:**

It is common for individuals under the age of 65 to consult neurologists for cognitive complaints ranging from subjective cognitive issues to mood disorders that affect cognitive function. The challenge is rule out attention deficit hyperactivity disorders, other psychiatric causes, and cognitive impairment.

In 2020, the Lancet Commission released a report analyzing 12 modifiable risk factors (MRFs) by age group. A recent study found that 6 of these factors, among others, were associated with the risk of Young‐Onset dementia: lower formal education, alcohol use disorder, social isolation, hearing impairment, diabetes, and depression.

This study aimed to identify cognitive risk factors in a young population with cognitive complaints.

**Method:**

Retrospective and descriptive study based on a neuropsychological evaluation database that focused on individuals aged between 40 and 60 who were assessed for cognitive complaints from July 2017 to July 2022. We collected demographic data, family history of neuropsychiatric disorders, 12 MRFs per the Lancet Commission, neuropsychological patterns, and diagnosis.

**Result:**

A total of 307 neuropsychological evaluations were analyzed, of which 63% were females. The diagnoses were categorized as mild neurocognitive disorder (MND) in 45% of cases, deficit in 32%, normal cognitive performance in 21%, and major neurocognitive disorder in 2%. The most frequent neuropsychological pattern was non‐amnestic MND, observed in 115 (37.5%) cases. In 125 (40.7%) cases, family history was reported; in 186 (63%) cases, depressive symptoms were observed according to the Beck Depression Inventory‐II, while physical inactivity was reported in 160 (68%) of cases, hypertension in 107 (35%) of cases, obesity in 83 (27%) of cases, and 59 (21%) of cases reported living alone. Notably, physical inactivity was associated with cognitive impairment (p 0.04), indicating that patients with physical inactivity are 2.2 times more likely to experience cognitive impairment compared to non‐sedentary patients.

**Conclusion:**

A notable number of individuals displayed a family history of cognitive impairment, as well as symptoms of depression and MRFs including hypertension and physical inactivity. Only physical inactivity was found to be a contributing factor to cognitive impairment, highlighting the significance of addressing lifestyle factors in maintaining cognitive health.

